# African HIV/AIDS Trials Are More Likely to Report Adequate Allocation Concealment and Random Generation than North American Trials

**DOI:** 10.1371/journal.pone.0003491

**Published:** 2008-10-22

**Authors:** Nandi Siegfried, Michael Clarke, Jimmy Volmink, Lize Van der Merwe

**Affiliations:** 1 Clinical Trial Service Unit, University of Oxford, Oxford, United Kingdom; 2 United Kingdom Cochrane Centre, Oxford, United Kingdom; 3 Faculty of Health Sciences, University of Stellenbosch, Cape Town, South Africa; 4 South African Cochrane Centre, Medical Research Council, Cape Town, South Africa; 5 Biostatistics Unit, Medical Research Council, Cape Town, South Africa; 6 Department of Statistics, University of Western Cape, Cape Town, South Africa; Harvard School of Public Health, United States of America

## Abstract

**Background:**

Adherence to good methodological quality is necessary to minimise bias in randomised conrolled trials (RCTs). Specific trial characteristics are associated with better trial quality, but no studies to date are specific to HIV/AIDS or African trials. We postulated that location may negatively impact on trial quality in regions where resources are scarce.

**Methods:**

1) To compare the methodological quality of all HIV/AIDS RCTs conducted in Africa with a random sample of similar trials conducted in North America; 2) To assess whether location is predictive of trial quality. We searched MEDLINE, EMBASE, CENTRAL and LILACS. Eligible trials were 1) randomized, 2) evaluations of preventive or treatment interventions for HIV/AIDS, 3) reported before 2004, and 4) conducted wholly or partly (if multi-centred) in Africa or North America. We assessed adequacy of random generation, allocation concealment and masking of assessors. Using univariate and multivariate logistic regression analyses we evaluated the association between location (Africa versus North America) and these domains.

**Findings:**

The African search yielded 12,815 records, from which 80 trials were identified. The North American search yielded 13,158 records from which 785 trials were identified and a random sample of 114 selected for analysis. African trials were three times more likely than North American trials to report adequate allocation concealment (OR = 3.24; 95%CI: 1.59 to 6.59; p<0.01) and twice as likely to report adequate generation of the sequence (OR = 2.36; 95%CI: 1.20 to 4.67; p = 0.01), after adjusting for other confounding factors. Additional significant factors positively associated with quality were an *a priori* sample size power calculation, restricted randomization and inclusion of a flow diagram detailing attrition. We did not detect an association between location and outcome assessor masking.

**Conclusions:**

The higher quality of reporting of methodology in African trials is noteworthy. Most African trials are externally funded, and it is possible that stricter agency requirements when leading trials in other countries and greater experience and training of principal investigators of an international stature, may account for this difference.

## Introduction

Good methodological quality is necessary to minimise bias in randomised controlled trials (RCTs) [1]. Previous studies have shown that certain trial characteristics are related to methodological quality. These include the disease category under study [2], [3], whether the trial was multi- or single-centred [2], [4], whether approval by an ethics committee was obtained [5], the type of intervention [6], the type of journal where the trial was reported [6], [7], inclusion of a clear definition of the primary outcome [4], and the source of funding [2], [8]

Commentators have drawn attention to the challenges of conducting high quality trials in resource-poor settings [9]–[11]. Within the HIV/AIDS field, non-African researchers conducting trials within Africa on behalf of external agencies have reportedly prioritised speed and efficiency over ethical approval and due process, raising questions regarding the quality of such trials [12]–[14]. However, evidence linking location with trial quality appears limited to case reports of individual studies. Therefore, our principal aim was to comprehensively evaluate whether the reported methodological quality of trials in Africa differs from that of trials conducted in a better-resourced region of the world, North America. Furthermore, given the concerns about location and trial quality in the HIV/AIDS field, we focused on HIV/AIDS trials conducted in both continents.

## Methods

### Identification of African trials

We have described our searches for the African trials extensively elsewhere [15]. Briefly, in the absence of any Africa-specific trials database, we searched the following databases to ensure adequate identification of all possibly relevant trials conducted in Africa and published before 2004: MEDLINE, EMBASE, the Cochrane Central Register of Controlled Trials (CENTRAL) and LILACS; with the assistance of an experienced information specialist.

EMBASE and LILACS were included to maximise the yield of trials from Francophone and Lusophone Africa preferentially reported in French or Portuguese-language journals. Our search strategy involved a combination of three main stages: to identify records pertaining to any aspect of the prevention or treatment of HIV or AIDS, conducted in any country in Africa, and which were likely to be randomised controlled trials (see Table 1). We searched the electronic databases in the second half of 2004, ensuring adequate capture of all publications up to 2003 by taking account of the lag between publication of the trial report and indexing in the database. If we identified a trial with only baseline results reported before 2004 we then searched for any future trial reports regardless if these were published after 2003. An epidemiologist fluent in Portuguese assessed the Portuguese abstracts identified from the LILACS database.

**Table 1:** Inclusion and exclusion criteria for randomized controlled trials in Africa.

	Inclusion criteria	Exclusion criteria
**Intervention**	Efficacy or effectiveness of HIV/AIDS-specific intervention on clinical outcomes and/or viral load, including:	Safety, acceptability and dose-finding trials with no measurement of efficacy or effectiveness, including:
	• pilot studies of efficacy;	• comparisons of doses of drugs as a prelude to investigating establishing efficacy (so-called ‘dose-finding’ trials)
	• comparisons of the pharmacokinetic activity of drugs *if* the effects on clinical outcomes and/or viral load are also known to have been measured;	
	• comparison of doses of drugs with established efficacy *if* the effects on clinical outcomes and/or viral load are also known to have been measured	
	Efficacy or effectiveness of non-HIV/AIDS specific intervention, but with a sub-group of at least 5% HIV-positive participants and a minimum of 10 such people, on clinical outcomes and/or viral load	Trials assessing preventive behavioural interventions in people who were not HIV- positive without measuring HIV incidence as an outcome
**Location**	All or some of the randomized participants were resident in Africa (includes multinational trials with recruitment in Africa)	Trials that randomized Africans living outside the continent, and no-one resident in Africa
**Participants**	Infected with HIV-1, HIV-2 or dually infected, or in the case of prevention trials, HIV-negative, but at risk of HIV	
**Trial Date**	Reported prior to 2004 (if preliminary data only, authors contacted for additional results)	

### Identification of North American trials

We replicated the above search methods to identify North American HIV/AIDS trials, substituting the location phase with terms and text specific to the North American continent. This was defined geographically as Canada, the United States (US) and Greenland excluding the Caribbean and Central America [16]. As the comparison was intended to be between poorly and richly resourced regions, Mexico was not included in our definition. We searched MEDLINE, EMBASE and CENTRAL in mid 2005.

### Trial eligibility and data extraction

For the African trials, one author checked each abstract against our eligibility criteria for randomised trials (Table 1). Two experienced hand-searchers also read all the retrieved abstracts to identify those that reported controlled trials, regardless of location or disease profile. This dataset was then checked by another author for eligible African randomized trials and compared with the first dataset for level of agreement. We obtained the full article for all records judged to be potentially eligible from both datasets or about which we were uncertain. Where necessary, translation from French was conducted by a researcher fluent in the language. One author read all potentially eligible articles, determined final eligibility and extracted data for each trial into a customised MS Access database. To capture trial quality, we used the domain approach recommended by Juni *et al.* [17]and appraised the adequacy of the generation of the random sequence and allocation concealment, the masking of assessors, and the degree of loss-to-follow-up or attrition in each trial (see Table 2 for definitions). We developed a hierarchical decision-making process for missing or unclear data. Multiple reports for a single trial were linked and data from all relevant reports was assessed.

**Table 2:** Definitions for quality domains.

**Domain**	Sequence of generation		
**Definition**	The means by which the sequence of interventions was created		
**Rating**	**Adequate**	**Inadequate** *	**Unclear** *
	Computer- or calculator-generated (includes minimisation and biased urn approaches); Random number tables; Coin toss; Throwing a dice; Drawing lots	Days of the week; Medical record numbers; Alternate days; Birth dates	The process is reported as ‘randomized’ but no details are provided regarding the method
**Domain**	Allocation concealment		
**Definition**	The means by which the intervention assignment is concealed before and including at the point of allocation		
**Rating**	**Adequate**	**Inadequate** *	**Unclear** *
	Central randomization with central office retaining schedule (accept report of centralized process as ‘adequate’); Independent 3^rd^ party (allocates intervention and retains schedule; allocator has no knowledge of patients); Different parties for randomisation and allocation clearly stated; Secure computer assisted method e.g. password protected files; Sequentially numbered, opaque, sealed envelopes; Serially numbered, identical containers, allocated sequentially	No separation between person generating sequence and the allocator e.g. the person tossing the coin allocates the participants; Computer programme with no details provided re protection and access	Only report ‘sealed envelopes’; The process is described but it is still not clear how the process worked; There is no report of the process.
**Domain**	Masking of assessors		
**Definition**	This describes whether the person assessing the primary outcome (e.g. lab analyst, clinician) was blinded to the intervention received by participants.		
**Rating**	**Adequate**	**Inadequate** *	**Unclear** *
	The assessor responsible for the primary outcome was clearly reported as being unaware of the treatments received. No assumptions will be made that this is the case if the trial is reported as ‘double-blind’, or ‘placebo-controlled’	The assessors are clearly reported as being aware of the treatment received e.g. the clinician dispensing the un-blinded intervention was also responsible for the outcome assessment.	Assessor blinding is not reported and it is not possible to ascertain whether the assessors were unaware of treatment.

* The categories of ‘inadequate’ and ‘unclear’ were collapsed to create a binary variable for each quality domain for use in the logistic regression: ‘adequate (1)’ versus ‘inadequate or unclear (0)’.

For the North American trials, the abstracts were read by the two trained hand-searchers and those they identified as randomised trials were further checked by NS for eligibility using the identical inclusion criteria for African trials substituting North America for Africa (Table 1). A consecutive sample of 500 of the records identified by the hand-searchers was checked by another investigator (JV) and we resolved differences by discussion. NS extracted key characteristics for each of the potentially eligible trial abstracts. We sought to create a random sample of the North American trials, at least as large as the African dataset, for our comparison. We randomly sorted these records using the [sort random]function in STATA 8 and then selected the first 150 of these for analysis. To confirm that the sample was broadly representative of the full dataset we compared key trial characteristics between those records included in the sample with those excluded from the sample. NS then conducted data extraction for the North American trials included in the random sample, as had been done for the African trials. As part of a capacity development initiative, two African research assistants conducted duplicate data extraction for 20% of these trials serving as an additional check.

### Statistical analysis

Statistical analysis was conducted in STATA 8. Dichotomous trial characteristics of African trials were compared with North American trials using univariate logistic regression. Results are presented as Odds Ratio (OR), the 95% Confidence Interval (CI) and the p-value for each cross-tabulation. Variables with more than two categories were collapsed into binary variables (see Table 3). All continuous variables were assessed for normality and compared using the Wilcoxon rank sum test (if the distribution was non-normal) or the Student's unpaired t test (if normally distributed). We transformed independent continuous variables with skewed distributions to normality for analysis.

**Table 3:** Collapse of variable categories into binary format and numeric codes.

Variable	Previous categories	Collapsed categories	Code
**Centre**	Single centre	Single centre	1
	Multicentre, single country	Multicentre	0
	Multicentre, multinational	Multicentre	0
**Randomisation type**	Blocked	Complex	1
	Blocked stratified	Complex	1
	Simple stratified*	Complex	1
	Simple	Simple	0
**Allocation concealment**	Adequate	Adequate	1
	Inadequate	Inadequate or unclear	0
	Unclear	Inadequate or unclear	0
**Random generation**	Adequate	Adequate	1
	Inadequate	Inadequate or unclear	0
	Unclear	Inadequate or unclear	0
**Blinding of assessors**	Yes	Yes	1
	No	No or unclear	0
	Unclear	No or unclear	0
**Local ethics approval**	Reported	Reported	1
	Not reported	Not reported or unclear	0
	Unclear	Not reported or unclear	0

* the code ‘simple stratified’ refers to randomisation described as ‘stratified’, but with no report of blocking.

Univariate logistic regression was conducted for each of the dependent quality variables against all independent variables [18]. The independent variables were those trial characteristics that have previously been shown to be associated with trial quality. For each quality variable, we created an initial multivariate logistic regression model and reduced it to a final model. The initial model contained all the independent variables with p-values<0.2 for the significance testing results from the univariate analyses (Hosmer and Lemeshow approach [18]) as well as plausible interaction terms (US government funding with pharmaceutical funding for allocation concealment) to address potential effect modification. We then used backwards stepwise selection with a maximum p-value set at 0.05 to select the independent variables to include in each final model while simultaneously avoiding possible colinearity between variables. As our principal aim was to test the effects of location on quality parameters, we retained location in the optimal final model regardless of statistical significance. As our assumptions about trial quality are dependent on the overall quality of the reporting in the articles describing the trials, we included a variable to indicate whether a flow diagram recommended by CONSORT was included in the report [19]. This served as a proxy measure of reporting quality, on the basis of previous research, [20] and was retained in the final model regardless of statistical significance. The likelihood ratio and significance values are presented for each model and the OR, 95% CI and the p-value are given for each independent variable. To assess the robustness of our final models, we also performed sensitivity analyses using different model-building strategies.

## Results

### Search yield and trial identification

#### African trials

The search yielded a total of 12,815 records (7,734 from MEDLINE; 4,594 from EMBASE; 440 from CENTRAL; and 47 from LILACS). From these, we identified 284 discrete potentially eligible records and after obtaining the full articles, identified 80 relevant African trials. The reviewers agreed on 91% of the eligibility of records, indicating very good agreement. No eligible trials were identified from the LILACS search.

Trials have been conducted in 18 countries in sub-Saharan Africa with no trials conducted in North Africa. Table 4 presents the country where each trial took place and whether the study was a single- or multi-centre trial. Of the ten multicentre, multinational trials which recruited participants in an African country, three also had sites in North America (two of these trials had sites in Canada and the third reported simply that they had a ‘North American’ site).

**Table 4:** African trials by country and single versus multi-centre status.

Country	Number of RCTs
**Single centre, single country**	**46**
Botswana	1
Burundi	1
Cameroon	1
Cote d'Ivoire	4
Ethiopia	2
Kenya	7
Malawi	4
Nigeria	1
South Africa	2
Tanzania	4
Uganda	10
Zaire	2
Zambia	4
Zimbabwe	3
**Multicentre, single country**	**24**
Cameroon	1
Cote d'Ivoire	2
Kenya	3
Malawi	2
Senegal	1
South Africa	5
Tanzania	2
Uganda	4
Zambia	2
Zimbabwe	2
**Multicentre, multinational** *	**10**

* includes Rwanda and Burkina Faso.

#### North American trials

**Figure 1:**
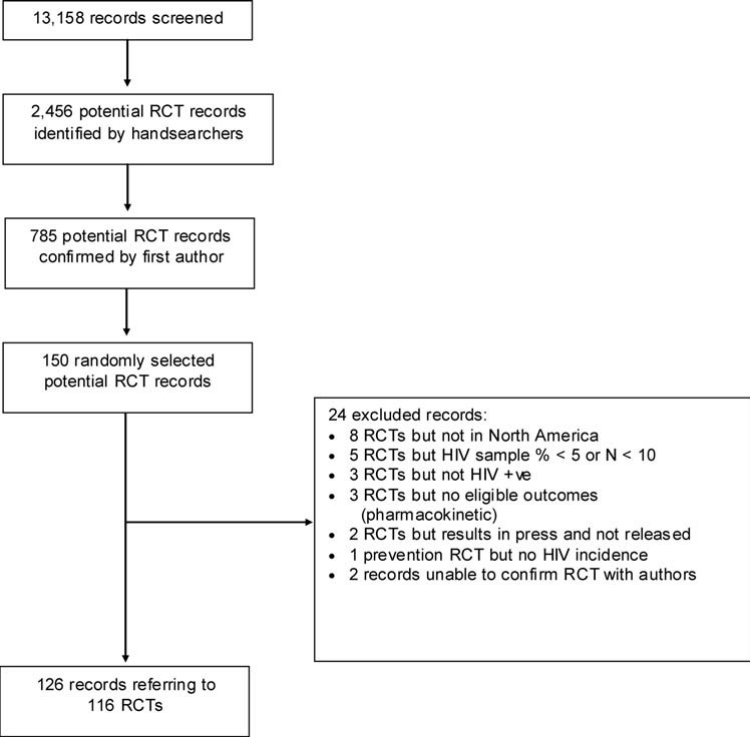
Flow diagram of eligibility selection process for North American trials.

The hand-searchers identified 2,456 records as randomized trials and 785 of these were judged eligible by the first author (Figure 1). We then selected the first 150 of the randomly sorted 785 records, which yielded 116 discrete North American trials. Ninety-six trials were based exclusively in the US, six exclusively in Canada, and 14 were multinational trials including sites either in Canada or the US, or both. No trials from Greenland were found. The distribution of key trial characteristics in the included sample was similar to that in those records that were excluded from the overall dataset (see Table 5), indicating a representative sample.

**Table 5:** Distribution of key characteristics in the overall North American dataset (785) and the random sample (150) of potentially eligible North American trials records.

Key characteristics	Sampled (N/150)	Not Sampled (N/634)	Sampled (%)	Not Sampled (%)	Fisher test p-value
**Prevention intervention**	15	79	10	12	0.48
**HAART as intervention**	30	163	20	26	0.17
**Behavioural intervention**	12	78	8	12	0.16
**Definitely in North America**	143	596	95	94	0.57
**Uncertain that HIV-related trial**	10	32	7	5	0.42
**Database(s) where record identified**	102	429	68	68	0.96*

* Null hypothesis: there was no difference between the number of records identified from each of the seven databases searched for the sampled and not sampled groups. The p-value is testing that the number of records identified in at least one of the seven different databases did not differ from that of at least one other database.

### Comparison of African and North American trials

#### Outliers and missing data

Of the 80 African and 116 North American trials, four African and one North American trial were identified as outliers due to their very large sample sizes. These five large trials were all community prevention trials with four using community cluster randomisation and the largest, a vaccine trial, randomized at an individual level but employing passive outcome reporting. These trials were sufficiently different from all the other trials in size for us to exclude them to avoid potentially obscuring the effect of interest in the regression analyses [21].

Information for all variables was complete for the remaining 191 records except for the variable: “Year of the start of the trial”. This was missing for 50 trials in total: 18% (14/76) of the African trials and 31% (36/115) of the North American trials. This is potentially an important variable because the time span for our study covers two decades and it is possible that more recent trials were more likely to conform to the conduct outlined in reporting standards such as CONSORT, which were first published in 1996 and revised in 2001 [22]. We found that the year of publication for the primary report was closely correlated with the year when the trial commenced for the 141 trials which contained complete information for both these dates (correlation coefficient = 0.89) and, so, we used the year of publication of the primary report as a proxy measure for the year in which the trial commenced in the full dataset of 191 trials. This ranged from 1984 to 2005 but was skewed by a North American trial (20 participants) published in 1984, five years before the next trial in the sample was published, in 1989 [23]. We excluded this trial from our detailed analyses, leaving 190 trials overall (80 African and 114 North American) for further analysis.

#### Association between location and reporting of trial characteristics

Preliminary overview of the trials revealed that the sources of funding for trials were heterogenous, with many trials bring funded by multiple agencies. As most North American (89% (102/114) and African trials (68% (52/76) were funded by either US government agencies, the pharmaceutical industry or a combination of these, we categorised funding to distinguish trials in two distinct ways: 1) receiving any US government funding versus not receiving any US government funding and 2) receiving any pharmaceutical industry funding versus not receiving any pharmaceutical industry funding.

Table 6 shows the comparison between the trial characteristics of the two locations for categorical variables. Overall, African trials were statistically significantly less likely than North American trials to assess a treatment intervention (OR = 0.17; 95%CI: 0.09;0.34; p<0.01), and statistically significantly more likely to be based in a single centre (OR = 2.40; 95%CI: 1.31;4.31; p<0.01), to report conducting an *a priori* power calculation based on a primary outcome (OR = 1.97; 95%CI; 1.09; 3.54), to contain a flow diagram as recommended by CONSORT (OR = 3.93; 95%CI; 1.97; 7.83; p<0.01), and to report adequate generation of the randomisation sequence (OR = 2.75; 95%CI: 1.43; 5.20) and adequate allocation concealment (OR = 3.75; 95%CI: 1.99; 7.05; p<0.01). North American trials were significantly more likely to have received funding from the US government (OR = 0.35; 95%CI: 0.19; 0.64; p<0.01).

**Table 6:** Trial characteristics by location of trial (N = 190).

Trial characteristic (variable)	Africa		North America		OR*	95% CI*	P value
	n/N	%	n/N	%			
**Intervention**							
Treatment	39/76	51	98/114	86	0.17	0.09; 0.34	<0.01
Prevention	37/76	49	16/114	14			
**Centre**							
Single centre	42/76	55	39/114	34	2.40	1.31; 4.31	<0.01
Multi-centre, single country & multinational	34/76	44	75/114	66			
**Type of randomisation**							
Simple	33/76	43	49/114	43	0.98	0.55; 1.76	0.95
Blocked and/or stratified	43/76	57	65/114	57			
**Primary outcome clearly defined**							
Yes, in trial report	61/76	80	77/114	67	1.95	0.98; 3.89	0.06
No, by hierarchy	15/76	20	37/114	33			
**Power calculation ** ***a priori***							
Yes	42/76	55	44/114	39	1.97	1.09; 3.54	0.03
No/Unclear	34/76	45	70/114	61			
**CONSORT flow diagram**							
Reported	31/76	41	17/114	15	3.93	1.97; 7.83	<0.01
Not reported	45/76	59	97/114	85			
**Allocation concealment**							
Adequate	39/76	51	25/114	22	3.75	1.99; 7.05	<0.01
Inadequate or not reported	37/76	49	89/114	78			
**Generation of random sequence**							
Adequate	31/76	41	23/114	20	2.75	1.43; 5.20	<0.01
Inadequate or not reported	45/76	59	91/114	80			
**Blinding of assessor**							
Yes	25/76	33	30/114	26	1.37	0.73; 2.59	0.33
No or unclear	51/76	67	84/114	74			
**Ethics approval**							
Local approval obtained	59/76	78	84/114	74	1.24	0.63; 2.45	0.54
No local approval reported	17/76	22	30/114	26			
**Funder: US Government**							
Any US government funding	24/76	32	66/114	57	0.35	0.19; 0.64	<0.01
No US government funding	52/76	68	48/114	43			
**Funder: Pharmaceutical**							
Any pharmaceutical funding	42/76	55	71/114	62	0.75	0.41;1.35	0.34
No pharmaceutical funding	34/76	45	43/114	38			

* Odds Ratio (OR), 95% Confidence Intervals (95%CI) and p values calculated using univariate logistic regression.

#### Association between location and trial sample size and year of publication

Distributions for the number of participants in both African and North American trials were positively skewed with many more small trials than large trials. African trials (median = 280; range: 16 to 2,219) were statistically significantly larger than North American trials (median = 101; range 12 to 2,493; p<0.01).

The year of publication for the primary report for African trials ranged from 1990 through to 2005, with the most frequent year of publication being 1999. For North American trials, the year of publication for the primary report ranged from 1989 through to 2005, with the most frequent publication year being 1998. Overall, the reports of African trials were published more recently (p<0.01) with a mean of 1999 for African trials and 1997 for North American trials.

#### Comparison between reporting of quality domains in African and North American trials

Univariate and multivariate regression analyses were conducted for the quality domains of allocation concealment, generation of the random sequence and assessor masking. The information reported on attrition for the primary outcome was inconsistent across the trial reports, meaning that the rate of attrition could not be calculated confidently for many trials and so we did not attempt comparative analysis for this.

#### Allocation concealment

Table 7 shows the results of individual tests of association between reporting of allocation concealment and each of the variables. It also summarises the final model; the four variables that jointly showed the strongest association with reporting of adequate allocation concealment were: where the trial was conducted, the inclusion or not of a CONSORT flow diagram, whether the trialists reported conducting an *a priori* power calculation based on the primary outcome, and whether complex randomisation (blocking, stratification or both) was used. After adjustment, trials conducted in Africa were independently associated with reporting of adequate allocation concealment. African trials were three times more likely to report adequate allocation concealment than North American trials (OR = 3.24; 95%CI: 1.59; 6.60; p<0.01). Year of publication, on its own, was strongly associated with allocation concealment, but not after adjusting for the variables mentioned earlier.

**Table 7:** Univariate & multivariate logistic regression of trial characteristics associated with adequate vs inadequate or unspecified allocation concealment (N = 190).

Variable	Univariate analysis				Logistic regression (final model) Likelihood Ratio *X* ^2^ (4) = 44.55; p<0.01			
	OR	L95%CI	U95%CI	P value	OR	L95%CI	U95%CI	P value
**Location**								
Africa	3.75	2.00	7.06	<0.01	3.24	1.59	6.59	<0.01
**Intervention**								
Treatment	0.28	0.15	0.55	<0.01	.	.	.	.
**Centre**								
Single	0.80	0.43	1.48	0.48	.	.	.	.
**Power calculation**								
Reported	3.95	2.09	7.49	<0.01	2.32	1.13	4.76	0.02
**CONSORT diagram**								
Reported	4.67	2.33	9.33	<0.01	2.25	1.02	4.93	0.04
**Primary Outcome**								
Defined	2.32	1.10	4.91	0.03	.	.	.	.
**Local ethics**								
Obtained	3.14	1.37	7.21	0.01	.	.	.	.
**Random type** *								
Complex	3.30	1.70	6.42	<0.01	2.51	1.17	5.37	0.02
**Random generation**								
Adequate	2.68	1.39	5.14	<0.01	.	.	.	.
**Assessor blinding**								
Yes	2.29	1.20	4.38	0.01	.	.	.	.
**US Government**								
Any funding	0.68	0.37	1.26	0.22	.	.	.	.
**Pharmaceutical**								
Any funding	1.48	0.79	2.76	0.22	.	.	.	.
**Log sample size**	4.25	2.25	8.02	<0.01	.	.	.	.
**Year primary report**	1.13	1.03	1.23	<0.01	.	.	.	.

* Random type categorised the random process as 1) simple or 2) complex (included blocking and/or stratification).

The quality of the journal where the report was published[7], as measured by whether or not the journal of the primary report was indexed in the Abridged Index Medicus (AIM) database, did not affect whether adequate allocation concealment was reported. To investigate whether reporting the year of the start of the trial was important, we repeated the analysis for only those trial reports where we had complete information for the year of the start of the trial (N = 141). The same four variables were jointly selected with the exception that the inclusion or not of the CONSORT flow diagram was no longer independently significant.

#### Random generation

Table 8 shows the results of individual tests of association between the reporting of generation of the random sequence and each of the variables. It also summarises the final model; the three variables that jointly showed the strongest association with reporting of generation of the random sequence were: where the trial was conducted, the inclusion or not of a CONSORT flow diagram and whether the trialists reported conducting an *a priori* power calculation based on the primary outcome. After adjustment for other variables, trials conducted in Africa were significantly more likely to report adequate generation than North American trials (OR = 2.36; 95%CI: 1.20; 4.67; p = 0.01). Trials in which the trialists reported conducting an *a priori* power calculation based on the primary outcome were also significantly more likely to report adequate generation compared with trials not reporting this, after adjusting for other variables. Year of publication was not independently associated with random generation.

**Table 8:** Univariate & multivariate logistic regression of trial characteristics associated with adequate vs inadequate or unspecified generation of the random sequence (N = 190).

Variable	Univariate analysis				Logistic regression (final model) Likelihood Ratio *X* ^2^ (3) = 15.1; p < 0.01			
	OR	L95%CI	U95%CI	P value	OR	L95%CI	U95%CI	P value
**Location**								
Africa	2.73	1.43	5.20	<0.01	2.36	1.20	4.67	0.01
**Intervention**								
Treatment	0.43	0.22	0.84	0.01	.	.	.	.
**Centre**								
Single	1.37	0.73	2.58	0.33	.	.	.	.
**Power calculation**								
Reported	2.46	1.29	4.70	<0.01	2.09	1.05	4.17	0.04
**CONSORT diagram**								
Reported	2.00	1.00	4.01	0.05	1.20	0.56	2.59	0.64
**Primary Outcome**								
Defined	1.97	0.90	4.28	0.09	.	.	.	.
**Local ethics**								
Obtained	2.31	1.00	5.34	0.05	.	.	.	.
**Random type** *								
Complex	1.99	1.02	3.88	0.04	.	.	.	.
**Allocation concealment**								
Adequate	2.68	1.39	5.14	<0.01	.	.	.	.
**Assessor blinding**								
Yes	2.15	1.10	4.19	0.03	.	.	.	.
**US Government**								
Any funding	0.79	0.42	1.49	0.46	.	.	.	.
**Pharmaceutical**								
Any funding	0.72	0.38	1.36	0.31	.	.	.	.
**Log sample size**	2.53	1.37	4.66	<0.01	**.**	**.**	**.**	**.**
**Year primary report**	1.05	0.96	1.14	0.26	**.**	**.**	**.**	**.**

* Random type categorised the random process as 1) simple or 2) complex (included blocking and/or stratification).

When considering only trials with complete information on the date of the start of the trial (N = 141), we found that the location of the trial was the only variable significantly associated with reporting of adequate generation of the random sequence, after adjusting for other variables. Whether the journal was indexed in AIM did not affect the results.

#### Masking of Assessors

Only one independent variable, type of randomisation, was significantly associated with reporting of masking of assessors of outcomes in the trials, with (OR = 2.33; 95%CI:1.19;4.57) and without (OR = 2.22; 95%CI:1.11;4.45) adjusting for possible confounders. Neither location nor inclusion of a CONSORT flow diagram were significantly associated with reporting of masking of assessors of outcomes in the trials, not individually, nor independently after adjusting for other variables.

#### Impact of the Principal Investigator

The Principal Investigator (PI) was clearly reported in very few trials (African: 30% (23/76); North American: 9% (9/114)). As most PIs were first authors of the primary report, we assumed the first author was the PI where this was unclear. We had insufficient information to code PI education or experience, or whether a statistician contributed to the report, but we explored whether there was a difference between the quality of those African trials led by a PI resident in Africa (according to their corresponding address) compared with African trials led by PIs from outside Africa. Twenty-nine percent (22/76) of African trials were led by PIs based in Africa, with no change in this trend over time. Of those African trials which reported conducting adequate allocation concealment, 31% (12/39) were led by an African PI. Of the 37 African trials reporting inadequate or unclear allocation concealment, 27% (10/37) were led by African PI. This was not a statistically significant difference (OR = 1.20; 95%CI: 0.40; 3.68; p = 0.72). Of the trials reporting adequate generation of the random sequence, 29% (9/31) were led by an African PI compared to 29% (13/45) of the trials reporting inadequate or unclear random generation. This was not a statistically significant difference (OR = 1.00; 95%CI: 0.32; 3.07; p = 1.00). We also explored whether North American PIs were associated with adequate allocation concealment and generation compared with non-North American PIs in the 76 African trials, and found no statistically significant differences.

## Discussion

To our knowledge, our study represents the first comparative analysis of the reporting of methodological quality of clinical trials in resource-rich versus resource-poor parts of the world. It has uncovered the noteworthy finding that the reports of trials done in a resource-poor setting show higher methodological reporting quality than trials done in well-resourced locations.

### Strengths and limitations

The strength of our study lies in its comprehensive search for African and North American trials across multiple databases [24], rigorous eligibility and data extraction processes conducted by more than one researcher [25], multivariate analyses to adjust for potential confounders and use of strict *a priori* criteria, based on previously reported research evidence, to determine the inclusion of potential confounders in our analyses. All regression analyses are highly dependent on the choice of variables for inclusion into the model, and possible residual confounding may be present especially due to issues of collinearity. We attempted to address this by consecutively adding or removing variables, and analysing subsets of individuals (extensive sensitivity analyses). This did not change the best selection of variables showing the robustness of the models for allocation concealment and generation of the random sequence. We did not convert continuous into categorical variables to avoid the serious biases that this might introduce [26]. However, some detail was inevitably lost when collapsing categorical into binary variables. For example, Schulz and colleagues [27], [28] pointed out that there may be differences, particularly in heterogeneity, between trials where allocation concealment was inadequate compared with trials where allocation concealment was unclear. However, less than five trials in both datasets combined were coded as ‘inadequately concealed’, making lack of retention of these three categories less likely to result in bias in this study.

As with all observational studies, our findings are limited in that the associations we found are not necessarily causally related. In addition, the data we included for each trial is entirely dependent on the quality of reporting for that trial and may not be a true reflection of the actual quality of the conduct of that trial. Most journals have specific limits on the number of words in an article and authors and editors may choose to reduce the length and detail of the methods section for a trial in order to leave adequate room for the results, discussion and conclusions. A consequence of this is that readers may be forced to regard the trial as being of a lesser methodological quality and, therefore, they might have less confidence in those results and conclusions. In order to establish causality, further research would first be required to compare trial reporting with actual trial conduct and then to compare these findings across locations. This would involve appraisal of the trial protocol and its amendments, observation of the trial conduct and appraisal of the final trial report. However, given that reports accessible in the public domain are the primary source of knowledge about the results of trials for policy-makers and clinicians, we believe that these are appropriate for assessing the quality of those trials used to influence policy and clinical decisions.

Throughout the research process, we based decisions regarding missing or unclear data on reasonable assumptions, but it is possible that measurement bias may have been introduced into the analysis, resulting in over- or under-estimates of the associations we found depending on the direction of the association in the misclassified trials [29]. The decision to restrict our searching to bibliographic databases was primarily pragmatic, leading to all but three of the included trials being published in journals (three African trials were unpublished and had been identified from news reports indexed in MEDLINE).We did not address impact factor directly due to the conflicting evidence regarding its utility for journal quality measurement [7], [30], [31]. Instead, we used a variable coding for whether a trial report included a CONSORT-recommended flow diagram as the measure of ‘reporting quality’ and were therefore able to adjust for this [20]. Furthermore, in the absence of any accepted, evidence-based classification of journal quality, we also included whether the journal for each trial's primary report was in the Abridged Index Medicus (AIM) in a sensitivity analysis. Inclusion in the AIM was likely to provide some indication of the status over time of a journal within the English-speaking world [32].

### Possible explanations and implications

The difference in the reporting of methodological quality of African trials compared with North American trials may not arise from a single factor, but from a combination of inter-related factors. Based on the characteristics of the trials included in our study and what is known from other research, a number of possible explanations for the observed difference may exist. We suggest some possible explanations here informed by our findings, while recognising that further research will be needed to assess these. Firstly, almost all the African trials were led and funded by organisations (governmental, non-governmental, and commercial) which operate in an international domain [15]. The rigorous process required to procure funding for such trials might make them more likely to be done by highly-skilled and experienced investigators. It is possible that locally driven and funded trials conducted in North America do not have to meet the same challenges of international peer review and intense competitive selection. If this is the case, a higher proportion of trials led by investigators with international reputations may account for the higher quality present in African trials. As all but two of the African trials received some funding from international agencies, it is also possible that those African principal investigators leading African trials were researchers familiar with the conduct of international trials and likely to have an international reputation.

Secondly, we showed that the presence of an *a priori* power calculation and the use of restricted randomization, were also significant factors in overall trial quality. These factors may indicate careful planning and a high level of trial expertise, such that the investigators leading African trials might be more likely to have epidemiological or statistical skills than the researchers who led the trials in North America. However, as noted in our Results section, we had insufficient information on the qualifications or experience of the PIs to test this hypothesis.

Thirdly, many African and North American trials involved more than one agency working collaboratively. A study of 235 gastroenterology trials found that multi-centre trials were of a higher quality than single-centre trials [2] with the authors suggesting that more researchers working on a trial ensures strict and careful planning and conduct of trials. In our study, African trials were more likely to be based in single centres, but in many of the African trials collaborating agencies and trial investigators were from different countries, with European or North American agencies working in partnership with African researchers [15]. In contrast, agencies collaborating in the North American trials were generally all based in the country where the trial was conducted, either the US or Canada. The challenges associated with working cross-culturally, especially with reference to African HIV/AIDS research, have been previously described and include tensions related to differential access to financial resources and facilities, different expectations of participation and of transfer of technology, of training opportunities and of credit for contributions [33]. We speculate that the cross-cultural collaboration in African trials, with all the associated competing interests, may create a working environment compelling investigators to work harder to achieve their aims, resulting in higher quality trials.

Lastly, a survey in 2000 of *The Lancet's* peer referees based in poor regions, concluded that researchers based in these regions believe that there is substantial editorial bias against their work [34]. In a study to evaluate whether publication of studies from poor countries is dependent on their quality, Yousefi-Nooraie and colleagues compared the methodological quality and significance of the results of trials from countries with different development status [35]. They found that overall, country income had a non-significant inverse association with the presence of randomization and a direct significant association with the use of blinding. The authors suggest that over time, authors from poor countries may be choosing to selectively report the studies that are larger, have less serious limitations, and contain positive and significant findings in international English language journals, because of the presumption that editors and reviewers will be biased against their nationality. Should such a bias exist, then it could contribute to the higher quality of the reporting of African trials since those African trials that are published will have been well-conducted and well-reported because these will have been key determinants in their selection for publication.

### Future research

Our findings indicate that location of a trial, or factors inherent in the location of a trial, may influence the reporting of trial quality. The findings support the need to plan trials carefully as evidenced by reporting of methodological quality being associated with an *a priori* sample size calculation, valid methods of randomisation, and clear and complete reporting of the trial as recommended by CONSORT. Difficulties associated with identifying trials and missing data will be reduced by prospective trial registration as advocated by the World Health Organization [36]. Prospective registration will facilitate further comparative studies such as this one in the future, as will closer adherence to the CONSORT standards for reporting trial results. Currently only one African country has a national prospective trials register and few African countries have legal imperatives to ensure prospective registration. Further research is required to delineate whether our finding is specific to the reporting of HIV/AIDS trials, trials in Africa compared to North America, or both. Better understanding of the factors associated with location will allow researchers, funding agencies, and others to address these factors more specifically when planning and reporting trials in future.
